# Cognitive Functions after Carotid Artery Stenting—1-Year Follow-Up Study

**DOI:** 10.3390/jcm11113019

**Published:** 2022-05-27

**Authors:** Magdalena Piegza, Izabela Jaworska, Jacek Piegza, Kamil Bujak, Paweł Dębski, Aleksandra Leksowska, Piotr Gorczyca, Mariusz Gąsior, Robert Pudlo

**Affiliations:** 1 Department of Psychiatry, Faculty of Medical Sciences in Zabrze, Medical University of Silesia in Katowice, 42-612 Tarnowskie Gory, Poland; pdebski@sum.edu.pl (P.D.); leksaleksa@wp.pl (A.L.); pgorczyca@sum.edu.pl (P.G.); rpudlo@sum.edu.pl (R.P.); 2 Department of Cardiac, Vascular and Endovascular Surgery and Transplantology, Silesian Center for Heart Diseases, Medical University of Silesia in Katowice, 41-800 Zabrze, Poland; isabelle_jaworska@yahoo.pl; 3 Palliative Medicine Department, St. Camillus Hospital, 42-600 Tarnowskie Gory, Poland; 4 Third Department of Cardiology, Faculty of Medical Sciences in Zabrze, Silesian Center for Heart Diseases, Medical University of Silesia in Katowice, 41-800 Zabrze, Poland; jacek.piegza@gmail.com (J.P.); kamil_bujak@o2.pl (K.B.); m.gasior@op.pl (M.G.)

**Keywords:** cognitive functions, carotid artery stenting, carotid artery stenosis, asymptomatic carotid stenosis, cognitive impairment

## Abstract

Background: The revascularization of carotid arteries minimizes the risk of future cerebral stroke and usually improves cognitive functions. The aim of this study was to assess changes in cognitive function and verify the hypothesis assuming an improvement of selected cognitive functions—psychomotor speed, visuospatial episodic memory, executive function and verbal fluency—in patients after carotid artery stenting during a 12-month follow-up. Methods: 47 persons subject to CAS, including 13 symptomatic persons, were examined before and 12 months after a procedure with a psychological test battery (digit symbol test—DS, Rey–Osterrieth complex figure test—ROCF, Wisconsin Card Sorting Test—WCST, letter verbal fluency—LVF). Sociodemographic data and clinical parameters were acquired from an author questionnaire. Results: The one-year follow-up, after the performed CAS procedure, demonstrated a significant improvement of psychomotor speed, visuospatial episodic memory, and executive function. No changes in the area of verbal fluency or decline in any of cognitive functions under analysis were observed. Conclusions: Carotid artery stenting improves cognitive functioning, both in the area of basic and more complex cognitive functions in persons with carotid atherosclerosis.

## 1. Introduction

The available literature shows that carotid revascularization prevents future cerebral stroke and, in certain carotid atherosclerosis patients, improves cognitive functioning. Following a proposal by the authors of the CREST-H (Carotid Revascularization and Medical Management for Asymptomatic Carotid Stenosis Trial—Hemodynamics) test, in case of this specific population of patients gaining cognitive benefits from recanalization of internal carotid arteries (ICA), the term “symptomatic carotid stenosis” should be redefined to include cognitive deterioration in the spectrum of symptoms [1]. According to this hypothesis, the improvement of neurocognitive functioning in symptomatic carotid stenosis patients should become another aim of recanalization procedures, in addition to cerebral stroke prevention.

Both carotid artery stenting (CAS) and endarterectomy (CEA) minimize the future risk of cerebral stroke and usually improve cognitive functioning. However, the available data differ slightly in this regard [2,3]. Most reports mentioned neurocognitive improvement after CAS [4,5,6,7,8,9,10,11,12,13,14,15,16,17,18,19,20], some recorded partial improvements [21,22,23], and one study recorded no changes [24]; in three studies, the deterioration of cognitive functions occurred [25,26,27].

Cognitive disorders in carotid atherosclerosis patients occurred, above all, as a result of: embolization, hypoperfusion, and diffuse cerebral white matter lesions [27]. On the other hand, mechanisms leading to neurocognitive deficits during revascularization procedures include: iatrogenic microembolization, so-called silent infarctions or atherosclerotic microembolisms, thrombosis and cerebral circulation disorders, impaired blood supply to the area supplied by the appropriate artery, and hyperperfusion. The comprehensive, long-term neurocognitive effect after revascularization comprises both factors related to the procedure itself and those dependent on the patient’s clinical condition. The observed improvement takes place as a result of the normalization of the cerebral blood supply and a reduction in embolization.

The aim of the present study is to assess the changes in the cognitive function of patients after carotid artery stenting and to verify the hypothesis of an improvement in selected cognitive functions—psychomotor speed, visuospatial episodic memory, executive function and verbal fluency—in patients after a carotid artery stenting procedure at 12-month follow-up. To this end, assessments of the functions mentioned above were performed before and 12 months after the stent implantation procedure at the Zabrze centre.

## 2. Material and Methods

The study included 47 persons hospitalized at cardiology wards of the Silesian Centre for Heart Diseases in Zabrze for the purpose of carotid artery revascularization over three consecutive years. Initially, 57 patients joined the study. However, 5 persons died before the second examination; 2 persons were excluded after the first examination due to a lack of significant stenoses in carotid arteries; for 1 person, the procedure was abandoned after effective pharmacotherapy; 1 person was subject to a cardiac surgery; 1 person resigned from participation in the project due to cooperation difficulties caused by lack of eyeglasses. The patients were qualified for carotid artery stenting on the basis of clinical interview and the degree of stenosis of the internal carotid artery, as demonstrated using the Doppler USG method. Significant stenosis, warranting hemodynamic intervention, was assumed to mean narrowed lumen of the vessel ≥70% in asymptomatic patients and ≥50% in symptomatic patients. Even in case of bilateral stenosis, which occurred seldom and was hemodynamically insignificant in one artery, the patient with a significant stenosis was subjected to the procedure. The study was attended by both symptomatic patients (13 persons) and asymptomatic patients (34 patients) (Table 1). The criterion of symptoms was undergoing an ischemic cerebral stroke and/or transient ischemic attacks (TIA) in the supply area of the narrowed artery over the last six months. In order to minimize the risk of embolization during the procedure, distal neuroprotection systems were applied in the form of filters. The study was conducted in two stages, i.e., before the angioplasty and carotid artery stenting, and 12 months after the procedure. The patients twice underwent a battery of cognitive tests, and the condition of carotid vessels was twice assessed via USG Doppler. Both the cognitive function assessment and the interpretation of results were performed by an experienced clinical psychologist. Eventually, only those persons who had positively completed both stages of the project, i.e., 47 people, were subject to statistical analysis. The criteria for inclusion were: age above 18 years, consented to participate in the study, qualification for carotid artery angioplasty surgery, no cognitive function disorders to a degree preventing performance of the tests. Due to large disproportion between the numbers of symptomatic and asymptomatic persons, two subgroups were not distinguished; the parameters under examination were analysed for all patients, who comprised one study group.

Psychomotor speed was measured using the digit symbol subtest (DS) from the WAIS (Wechsler Adult Intelligence Scale) test. The proper performance of this test relies on visual and motor processing under time pressure, assisted by incidental learning. The duration of a test is 90 s; 1 point is awarded for each correctly performed task (for a maximum of 93 points). Raw scores are transformed in accordance with the age norm table into calculated results (CR), falling within the range of 1–19 [28].

Rey–Osterrieth complex figure test (ROCF) was used to assess visual memory and spatial functions. A figure consists of 18 parts, each with the same value and weight, i.e., the obtained score directly depends on the number of the reproduced parts of the pattern. The maximum score to be obtained is 36. It was assumed that the indicators of performance for both a copy and a reproduction from memory was the number and quality of the recreated parts, and the score was obtained on this basis. The relation between the parameters under analysis and the time necessary to perform the copy and reproduction of the figure was not examined [29].

Executive functions were measured using the Wisconsin Card Sorting Test (WCST), valuable in the diagnosis of neuropsychological cognitive activity. It consists of 2 decks of 64 cards each. A patient must match each card from a deck to one of four model cards, following the rule of application they have determined themselves on the basis of the examiner’s reaction. Initially, this test was created in order to assess the ability of abstract thinking and changes in cognitive strategies in response to changing conditions of the environment. Therefore, it may be treated as a measure of “executive functions”, requiring the ability to develop and maintain an appropriate problem-solving strategy under conditions of changing stimuli in order to achieve this goal. WCST requires strategic planning, an organized search, and the utilization of feedback from the environment in order to change cognitive attitudes, the orientation of behaviour to achievement of goals, and modification of impulsive reaction. Raw scores are transformed into standardized results on the basis of tables for appropriate age categories [30,31].

Assessment of verbal fluency involved the letter verbal fluency test (LVF). Letter verbal fluency measures the speed of word production based on a phonological cue. It consists of uttering as many words as possible from a given category (semantic fluency) or starting with a specific letter (phonemic fluency) over a specific time. The authors of the present study focused on phonemic fluency. This task engages the functions of attention and memory, as well as executive and language functions. Disturbance of any of these functions may affect verbal fluency. We treated words repeating within one sample as perseverations, and we treated interpolations, or words starting with a different letter than given in the sample as intrusions [32].

The sociodemographic data and selected clinical parameters were collected using a specially developed questionnaire. This information was confronted with the data included in the patient’s discharge.

Categorical variables are presented as numbers of patients and percentages. Continuous variables are expressed either as mean and standard deviation (if normally distributed) or as median and interquartile range. The normality of data distribution was assessed with the Shapiro–Wilk test. The differences in the results of the neuropsychological tests at baseline and follow-up visits were compared using paired Student’s *t*-test or Wilcoxon signed-rank test where appropriate and depicted as box plots. The level of statistical significance was set at *Benjamini–Hochberg adjusted p* < 0.05 (false discovery rate, *FDR*). Statistica version 13.3 (TIBCO Software, Palo Alto, CA, USA) was used for all statistical analyses.

The study was conducted according to the guidelines of the Declaration of Helsinki and was approved by the Bioethical Commission of the Medical University of Silesia, L.dz.NN-6501-132/07. Written informed consent was obtained from all participants.

## 3. Results

The study eventually involved 47 people aged 58 to 68, 66% of whom were men. The criterion of symptoms was met by 13 persons. Full sociodemographic characteristics and selected clinical parameters of the described group are presented in Table 1 (Table 1).

**Figure 1:**
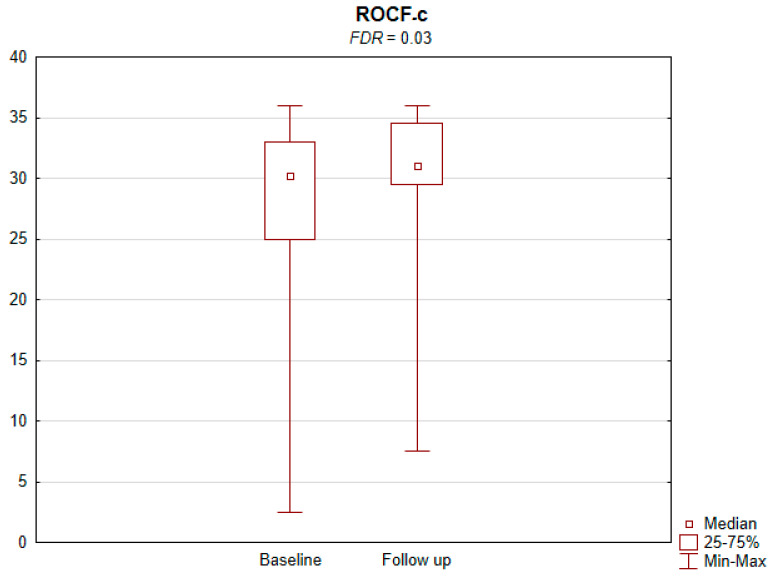
Rey–Osterrieth Complex Figure copy (ROCF-c) before and after the intervention. *FDR*—False Discovery Rate.

**Figure 2:**
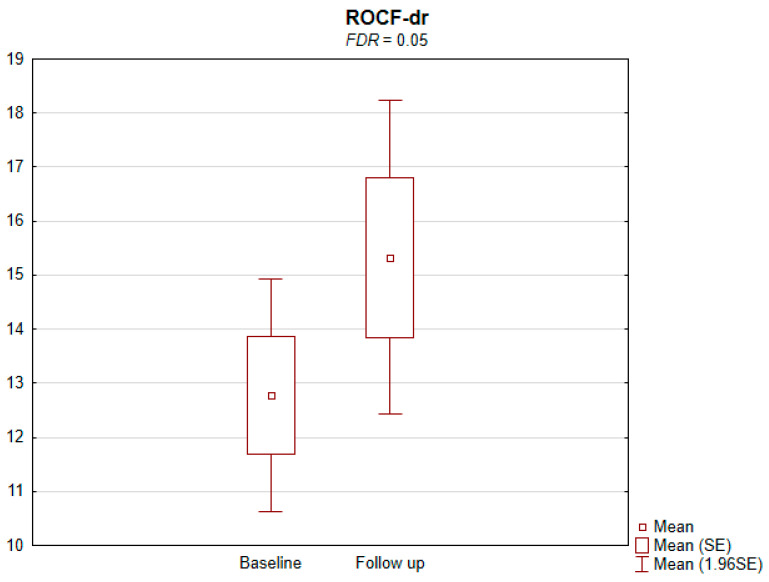
Rey–Osterrieth complex figure delayed recall (ROCF-dr) before and after the intervention. *FDR*—false discovery rate.

**Figure 3:**
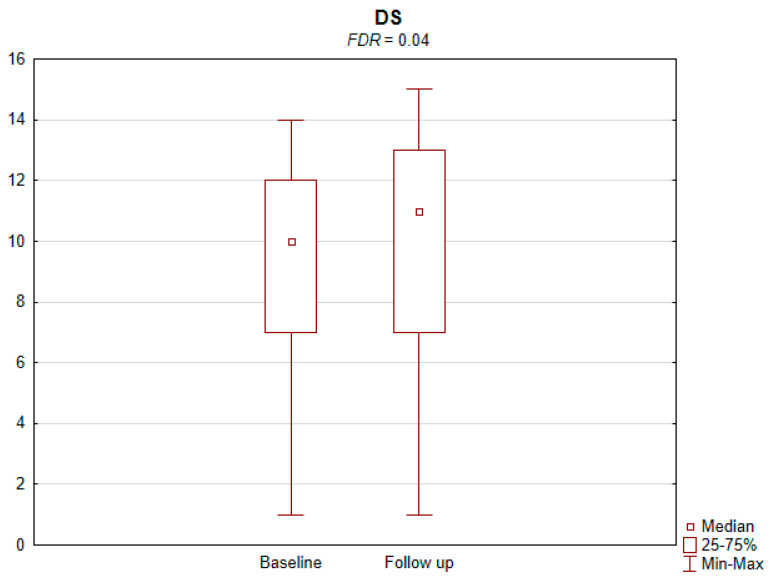
Digit symbol test (DS) on Wechsler Adult Intelligence Scale before and after the intervention. *FDR*—false discovery rate.

**Figure 4:**
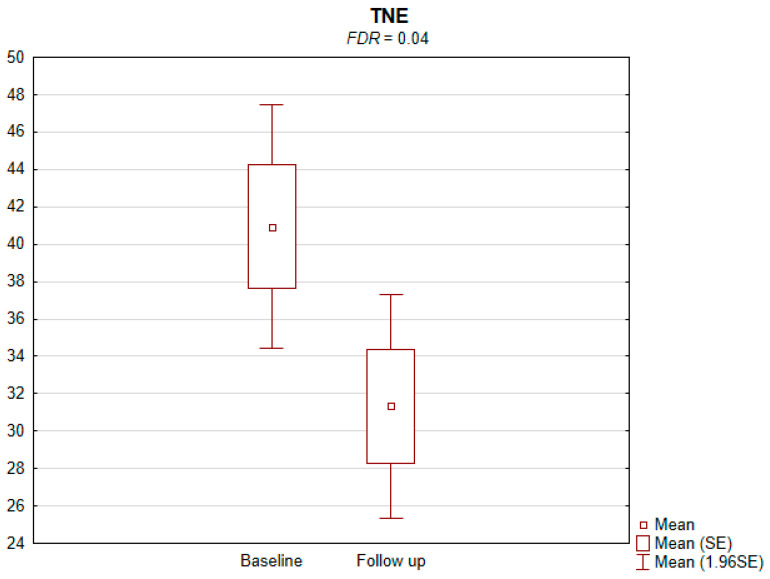
Total number of errors (TNE) in Wisconsin Card Sorting Test before and after the intervention. *FDR*—false discovery rate.

**Figure 5:**
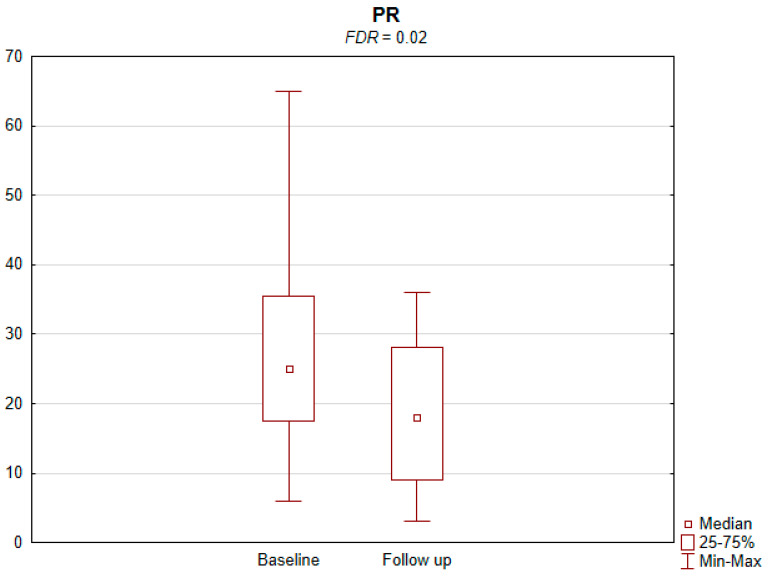
Perseverative responses (PR) in Wisconsin Card Sorting Test before and after the intervention. *FDR*—false discovery rate.

**Figure 6:**
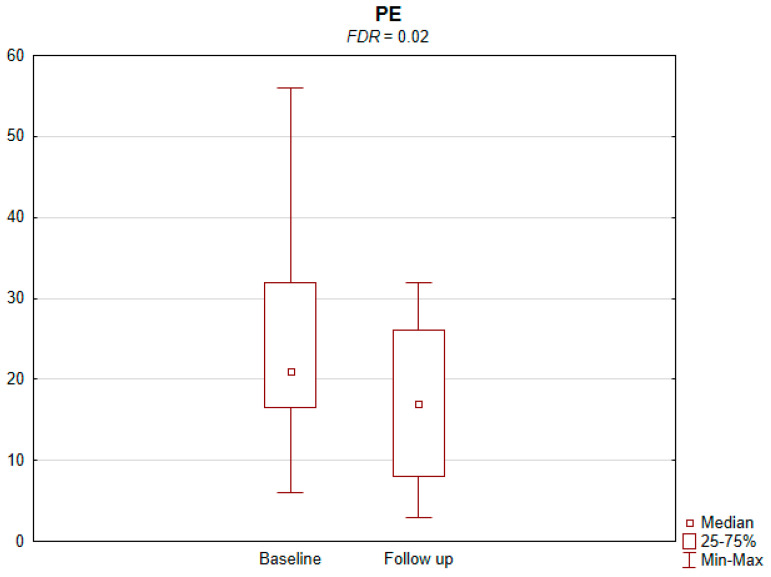
Perseverative errors (PE) in Wisconsin Card Sorting Test before and after the intervention. *FDR*—false discovery rate.

**Figure 7:**
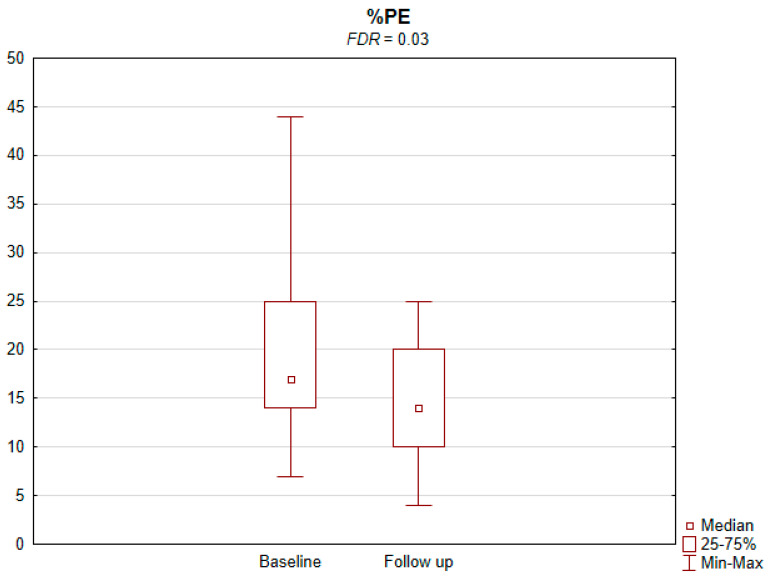
Percentage of perseverative errors (%PE) in Wisconsin Card Sorting Test before and after the intervention. *FDR*—false discovery rate.

**Figure 8:**
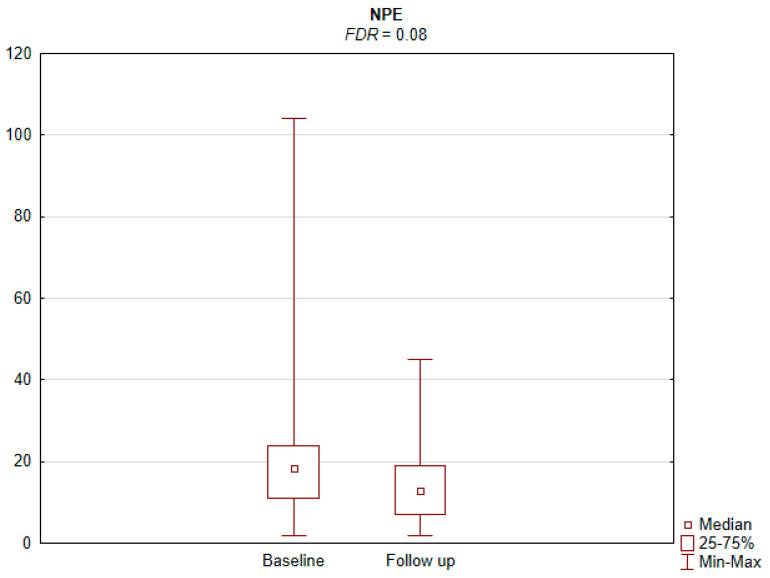
Non-perseverative errors (NPE) in Wisconsin Card Sorting Test before and after the intervention. *FDR*—false discovery rate.

**Figure 9:**
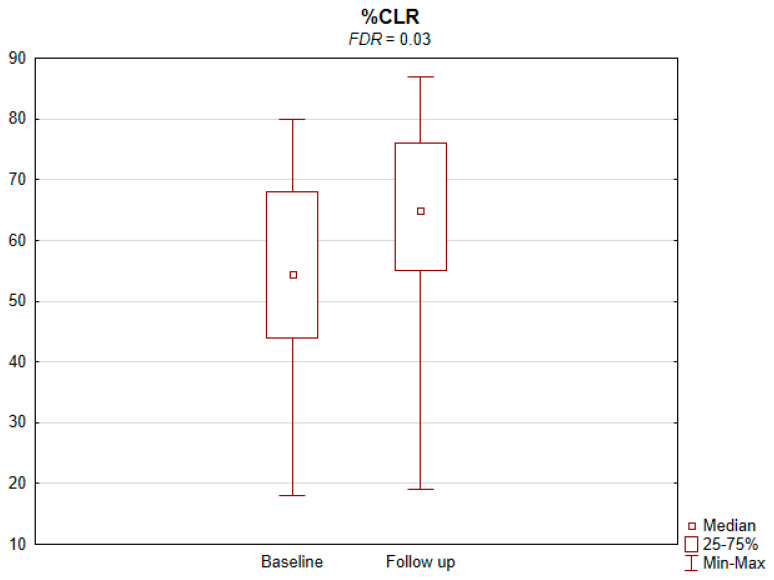
Percentage of conceptual level responses (%CLR) in Wisconsin Card Sorting Test before and after the intervention. *FDR*—false discovery rate.

The results of the conducted study demonstrate that persons subject to carotid artery revascularization showed a significantly improved performance of cognitive functions important from the viewpoint of everyday life. Simultaneously, no decline in any of the cognitive functions under analysis was observed upon a performed procedure (Table 2). As shown by the compilations of ROCF test results, visual–spatial abilities in patients after a CAS procedure significantly improved. A slight but significant improvement in the quality of task performance, which involved copying complex visuospatial material, was observed in the persons under examination, indicating an increase in the patient’s functioning in the areas of attention, perceptive structurization, as well as visual–motor control (*FDR* = 0.03; Figure ROCF-c (Figure 1)). There was also a definite improvement in the performance of delayed free recall of previously copied visuospatial information (*FDR* = 0.05; Figure ROCF-dr (Figure 2)). On the other hand, significant improvements in the performance of digit symbol test (DS) tasks suggested an increased efficiency in the area of psychomotor speed, which may translate to increased learning ability and improved visual–motor coordination *(FDR* = 0.04; Figure DS. (Figure 3)). The examined persons also improved their results in the Wisconsin Card Sorting Test (WCST). By analysing the differences in the range of results obtained before and after the intervention, a significant decrease in erroneous responses (TNE) was observed (*FDR* = 0.04, Figure TNE (Figure 4)). Sharp decreases were recorded for a number of perseveration responses (PR) (*FDR* = 0.02; Figure PR (Figure 5)) and perseveration errors (PE) (*FDR* = 0.02; Figure PE (Figure 6)). Furthermore, a significant decrease in the number of perseveration errors made was observed in the percentage expression of the results (%PR) (*FDR* = 0.04; Figure 7). The decline in the number of non-perseveration errors (NPE) (*FDR* = 0.08; Figure NPE (Figure 8)) became also close to achieving statistical significance. Simultaneously, a significant increase was recorded for a number of conceptual responses, considering the percentage expression of the results (%CLR) (*FDR* = 0.03; Figure %CLR (Figure 9)).

On the other hand, no significant changes in the area of language functions measured by the LVF test were observed (Table 2).

## 4. Discussion

Information from different centres shows that CAS is related to a higher frequency of occurrence of cerebral microembolisms in comparison with CEA [27,33,34,35]. Both CAS and low presurgical cerebral perfusion are risk factors for large-scale infarction occurring through the embolization mechanism related to the treatment procedure. The range of the ischemic area significantly affects cognitive functioning. Some researchers claim that, although CAS-related silent brain infarctions are described more frequently than in case of CEA, their effect on cognitive functions should not be overestimated; therefore, they suggest further research [35] (cognitive sequalae from procedure-related silent ischemic lesion)**.** Other scholars recommend considering the assessment of the neurocognitive condition in every patient subject to carotid artery revascularization [34]. Due to the fact that changes in the cognitive functioning of a patient after CAS are difficult to predict, different neuroprotective systems are used to minimize the risk of microembolization [16,17,27,33,34,36]. In our study, distal neuroprotective systems in the form of filters were also applied to all patients.

The results of our study show that persons subject to carotid artery stenting experienced a significant increase in visual–spatial abilities, as observed in the Rey–Osterrieth complex figure test (ROCF). Moreover, a clear increase in the correctness of performance of a task engaging visual memory was observed. Improvement in this area, 6 months after the procedure, was also recorded by Kougias et al. [33]. They assessed cognitive functions before 6 weeks and 6 months after the CAS (*n* = 29) or CEA (*n* = 31) in patients with severe asymptomatic stenosis in ICA ≥ 80%. They demonstrated an improvement of visual and verbal memory, attention functions, the speed of cognitive processes, executive and motor functions, memory, visual–spatial abilities in subsequent measurements in comparison with the first pre-procedure measurement. Furthermore, they proved the prevalence of CAS over CEA in improving the speed of cognitive processes, as well as executive and motor functions [33].

The increased efficiency in the area of psychomotor speed, obtained by our patients in the digit symbol Test (DS), reveals an improvement in the ability to absorb material and of the visual–motor coordination. The same conclusions were drawn by other researchers, emphasizing the prevalence of a group of patients subject to CAS over patients after endarterectomy (CEA) in memory and concentration tests, although improvement occurs in all researched persons after revascularization interventions in the carotid artery area, regardless of the method [36]. Similarly, another study of symptomatic patients after a cerebral infarction (*n* = 579), subject to CAS, observed an improvement in cognitive functions as measured with MoCA and MMSE tests 6 months after the procedure. These parameters remained unchanged or improved further during a 3-year follow-up [5]. The improvement of neurocognitive functioning after CAS in symptomatic patients with carotid artery stenosis in long-term observation is supported by findings from many studies [5,8,10,16].

Different conclusions were drawn by Chinese researchers who recorded no improvement in the digit span subtest from the MoCA (Montreal Cognitive Assessment) scale and quick recreation in asymptomatic patients with unilateral stenosis of at least 70% in ICA, subject to CAS. On the other hand, they noticed an improvement in the MMSE (Mini-Mental State Examination) score and verbal memory tests, as well as postponed memory 3 months after CAS. However, it should be noted that this study only covered 16 people [21].

The participants of our study also improved their results in the Wisconsin Card Sorting Test (WCST). The data obtained in this test indicate a decline in errors made by the researched group in the decision-making process and an increase in their ability to understand the changing rules of a task. They directly translate to the increased efficiency of executive functions, which are necessary to initiate deliberate activities and plan and maintain the direction of research. The improvement in health condition is an area of flexibility of action that exercises increased control of cognitive functions. This indicates an improvement in the ability to receive and analyse information, understand the information’s context, change the criterion of action in response to negative reinforcement, remember the previous criterion of action, or refuse certain criteria of reaction through experience-based reasoning.

Grunwald et al. examined asymptomatic, right-handed patients with arterial hypertension (*n* = 41) before and 3 months after CAS, using tests assessing the efficiency of performance functions (trail-making Test, labyrinth test, symbol–figure test, letter stroking) and tests of memory functions (tests of digit repeating, verbal, visual, delayed memory, latent learning), as well as MMSE and BDI (Beck Depression Inventory). The patients showed a significant improvement in the area of cognitive functions and everyday activity, no changes in the area of memory and verbal functions, and the results proved unrelated to the severity and size of the carotid stenosis or gender and age. Additionally, persons (34.2%) in whom changes perceptible in the DWI (cerebral diffusion weighted magnetic resonance imaging—MRI-DWI), evidencing the presence of microembolisms, were detected two days after the procedure, achieving similar results in cognitive tests after CAS for persons in whom such changes were not observed [4].

A Taiwanese study showed that carotid atherosclerosis for more than 10 years is related to the estimation of functional connectivity (FC) changes in specific cerebral structures in patients with unilateral stenosis in ICA after the stenting, as well as their effect on cognitive functions. This is the first project of this type, focusing on the compensative adaptation of the nervous system to changing conditions in patients with carotid stenosis. Researchers demonstrated that the irregularities of FC were connected with inferior cognitive parameters, especially regarding memory and executive functions, and showed a tendency to improve after CAS. Lateralization, as a compensative adaptation change, occurred in patients subject to CAS, as opposed to healthy persons from the control group [14]. The current clinical search on indicators enabling the prediction of the neurocognitive effects after CAS is represented in studies by Tani M. et al., who analysed eight patients with unilateral stenosis of ICA before and 6 months after the stenting, using rs-fMRI (resting-state functional MRI), a battery of neuropsychological tests, and DMN (default mode network). DMN was used to map every patient on the basis of an independent parameter obtained from rs-fMR. The relation between FC from DMN and changes in cognitive functions after CAS were examined. A negative correlation between DMN and the superior and middle–frontal gyrus was demonstrated in the area of working memory after CAS with changes in FC. The output FC between those cerebral regions was positively correlated with an improvement of working memory after the procedure. On the basis of these results, a hypothesis was advanced that a pre-reperfusion FC assessment may predict post-reperfusion changes in the area of working memory in patients treated for unilateral stenosis of ICA [18].

Other researchers also confirmed the significant improvement of executive functions and memory after vascular interventions in patients with carotid atherosclerosis [37,38,39,40,41,42].

No decline in any of the cognitive functions under analysis after a performed CAS procedure was observed in the patient population under our examination. Similar relations are also described by other researchers who did not record any decline in any area of cognitive functioning after a carotid artery stenting procedure [23,24,43].

No significant changes were demonstrated in the area of verbal fluency during one-year follow-up of our patients examined using the letter verbal fluency (LVF) test. The quickness and fluency of remembering and uttering words did not change after CAS, which is also reflected in the previously cited studies by other researchers [4,24,41].

Cognitive deterioration due to hypoperfusion was named by Bowler JV as “vascular cognitive impairment” [44]. Currently, vascular cognitive impairment refers to all forms of cognitive disorder associated with cerebrovascular disease and is common both after stroke and in asymptomatic individuals presenting with dementia symptoms. Vascular pathology frequently coexists with neurodegenerative pathology, resulting in mild cognitive impairment (MCI) or dementia [45]. The embolism caused by carotid artery stenosis can result in multiple infarcts, which lead to vascular cognitive impairments and vascular dementia (VD). CAS, by restoring perfusion in the stenosed area of the cortex, improves cognitive functions, and thus reduces the risk of developing VD [10,46]. Other publications report that carotid atherosclerosis promotes the transformation of MCI to dementia; in particular, symptomatic carotid artery atherosclerosis is a well-documented risk factor for developing MCI and VD [47]. In a Chinese study, CAS improved cognitive function in patients with carotid stenosis and MCI; the increase in MMSE and MOCA scores 6 months after the intervention was closely related to an improvement in cerebral perfusion [12]. Further studies are needed to evaluate the long-term effect of CAS on cognitive function in these patients and to establish its preventive role in the development of vascular dementia [12]. Listing the above facts and taking into account the results of our study, we can hypothesise that CAS protects against the development of VD by reducing the risk of stroke and improving cognitive parameters. However, this hypothesis requires further research.

Both our study and most previously published scientific reports show that one should cautiously consider the possibility of treatment for the impairment of cognitive functions, both in asymptomatic and symptomatic carotid atherosclerosis patients, as a “clinical symptom” of carotid artery stenosis, the presence of which may prejudge a decision to perform angioplasty and stenting in such patients. According to this assumption, the minimization of cognitive deterioration might become another aim of carotid artery revascularization, aside from the sole aim so far—cerebral stroke prevention in relation to a special group of patients, selected on the basis of peculiar clinical indicators with the prospect of a favourable effect of the procedure on neurocognitive functioning. Why do some parameters improve, while others do not? Does it depend on the representation of a given function in the central nervous system upon restoring better hemodynamic conditions, the assumed criteria and selection of neurocognitive tests, or other clinically significant factors? These questions still remain to be answered and need to be taken into consideration in the following studies.

## 5. Limitations of the Study

The main limitation of this study is the relatively small number of patients subject to CAS, even though a larger population of patients seldom appears in the available literature. We also did not include a control group. A group of healthy people and group of patients who do not undergo CAS would be beneficial for further studies. Moreover, an overly significant disparity between the number of symptomatic and non-symptomatic persons prevented an analysis of the researched cognitive parameters broken down into two groups. In the future, one should increase the size of the population under examination and develop the methodologies of constantly emerging, newer variants of neurocognitive tests performed with the aid of a computer. We should also note that the obtained results might come from test–retest effects. To weaken these effects, we conducted our study with a one year break between examinations. In future research, cognitive tests with alternative versions may be used. Furthermore, cognitive skills were measured with the use of only simple, single tests and, because of this, should be taken into consideration with caution. In addition to vascular and neurocognitive diagnosis, it would also be useful to assess the functional changes in the central nervous system, applying state-of-the-art neuroimaging methods using MRI.

## 6. Conclusions

1. In persons subject to carotid artery stenting, an improvement was achieved in several cognitive functions, such as psychomotor speed, visuospatial episodic memory, and executive function. No significant changes in the area of verbal fluency were recorded 12 months after the procedure.
2. No decline in any of the cognitive functions under analysis was observed after a performed CAS procedure during one-year follow-up.

## Figures and Tables

**Table 1:** Baseline characteristics.

Variables	All Patients (*n* = 47)
Sex (male)	31 (66.0%)
Age (years)	65 (58–68)
Level of education	
Primary	9 (20.0%)
Vocational	8 (17.8%)
Secondary	18 (40.0%)
Higher	10 (22.2%)
Employment	
Unemployed	1 (2.8%)
Professionally active	3 (8.3%)
Pensioner	32 (88.9%)
Marital status	
Single	3 (8.3%)
Married	27 (75.0%)
Widowed	6 (16.7%)
Mental disorders	2 (5.6%)
Previous stroke	12 (27.3%)
Previous TIA	1 (2.3%)
Previous CAS	1 (2.4%)
Previous CAD	32 (72.7%)
Previous PCI	15 (35.7%)
Previous CABG	5 (11.9%)
Malignancy	3 (6.8%)
Time until follow-up visit (days)	369 (327–448)

Categorical variables are shown as number of patients (percentage). Continuous variables are presented as median (interquartile range). TIA—transient ischemic attack, CAS—carotid artery stenting, CAD—coronary artery disease, PCI—percutaneous coronary interventions, CABG—coronary artery bypass grafting.

**Table 2:** Changes in cognitive functions assessed for the results obtained in the first and second measurements.

	Baseline	F-UP	*p*	*FDR*
ROCF-c	30.25 (25–33)	31 (29.5–34.5)	0.02	0.03
ROCF-dr	12.8 ± 5.9	15.3 ± 7.8	0.006	0.05
DS	31(20–38)	37 (22–42)	0.02	0.04
DS-c	10 (7–12)	11 (7–13)	0.04	0.07
LVF	43.0 ± 13.6	44.6 ± 15.4	0.36	0.38
LVF-P	1 (0–2)	1 (0–1)	0.36	0.40
LVF-I	0 (0–0)	0 (0–1)	0.97	0.97
NTP	128 (108.5–128)	121 (93–128)	0.08	0.11
NCS	76.0 ± 11.0	78.8 ± 11.5	0.24	0.29
TNE	40.9 ± 18.2	31.3 ± 16.7	0.008	0.04
TNE-SR	107.8 ± 11.3	114.7 ± 10.9	0.003	0.05
TNE-C	68 (47–87)	84 (66–91)	0.01	0.03
%E	33.7 ± 12.4	27.4 ± 12.5	0.01	0.02
PR	25 (17.5–35.5)	18 (9–28)	0.01	0.02
PR-SR	104 (95–113)	110 (101–121)	0.01	0.02
PR-C	67 (31–81)	81 (55–93)	0.009	0.03
%PR	19.5 (15–27.5)	15 (10–22)	0.006	0.04
PE	21 (16.5–32)	17 (8–26)	0.01	0.02
PE-SR	107 (95–115)	110 (102–122)	0.004	0.05
PE-C	68 (37–84)	75 (55–93)	0.02	0.04
%PE	17 (14–25)	14 (10–20)	0.006	0.03
%PE-SR	107 (95–114)	111 (102–123)	0.003	0.10
%PE-C	68 (37–82)	77 (55–94)	0.01	0.03
NPE	18.5 (11–24)	13 (7–19)	0.053	0.08
NPE-SR	106 (98–114)	110 (101–117)	0.052	0.08
NPE-C	66 (45–82)	75 (53–87)	0.08	0.11
%NPE	14.5 (9–18.5)	11 (7–15)	0.15	0.19
CLR	64 (56–72)	69 (60–78)	0.15	0.20
%CLR	54.5 (44–69)	65 (55–76)	0.01	0.03
%CLR-SR	107.7 ± 11.1	114.3 ± 12.5	0.009	0.04
%CLR-C	66 (47–87)	84 (68–93)	0.009	0.03
NCC	5 (3–6)	6 (3–6)	0.35	0.40
TC1sc	12 (11–21.5)	12 (11–31)	0.26	0.3
FMS	1 (0–2)	2 (0–3)	0.38	0.39

*FDR*—false discovery rate, ROCF-c—Rey–Osterrieth complex figure: copy, ROCF-dr—Rey–Osterrieth complex figure: delayed recall, DS—digit symbol test, DS-c—digit symbol test: convert, LVF—letter verbal fluency, LVF-P—letter verbal fluency: perseveration, LVF-I—letter verbal fluency: intrusion, NTP—number of tests performed, NCS—number of correct sorts, TNE—total number of errors, TNE-SR—total number of errors: standardised results, TNE-C—total number of errors: centile, %E—percentage of errors, PR—perseverative responses, PR-SR—perseverative responses: standardised results, PR-C—perseverative responses: centile, %PR—percentage of perseverative responses, PE—perseverative errors, PE-SR—perseverative errors: standardised results, PE-C—perseverative errors: centile, %PE-SR—percentage of perseverative errors: standardised results, %PE-C—percentage of perseverative errors: centile, %PE—percentage of perseverative errors, NPE—non-perseverative errors, NPE-SR—non-perseverative errors: standardised results, NPE-C—non-perseverative errors: centile, %NPE—percentage of non-perseverative errors, CLR—conceptual level responses, %CLR—percentage of conceptual level responses, %CLR-SR—percentage of conceptual level responses: standardised results, %CLR-C—percentage of conceptual level responses: centile, NCC—number of categories completed, NCC-C—number of categories completed: centile, TC1sc—trials to complete the first category, TC1sc-C—trials to complete the first category: centile, FMS—failure to maintain set.

## Data Availability

Data presented in this study are available on reasonable request from the corresponding author.
